# Beta-Arrestin Functionally Regulates the Non-Bleaching Pigment Parapinopsin in Lamprey Pineal

**DOI:** 10.1371/journal.pone.0016402

**Published:** 2011-01-31

**Authors:** Emi Kawano-Yamashita, Mitsumasa Koyanagi, Yoshinori Shichida, Tadashi Oishi, Satoshi Tamotsu, Akihisa Terakita

**Affiliations:** 1 Department of Biology and Geosciences, Graduate School of Science, Osaka City University, Osaka, Japan; 2 Department of Biophysics, Graduate School of Science, Kyoto University, Kyoto, Japan; 3 Nara Saho College, Nara, Japan; 4 Graduate School of Humanities and Sciences, Nara Women's University, Nara, Japan; Dalhousie University, Canada

## Abstract

The light response of vertebrate visual cells is achieved by light-sensing proteins such as opsin-based pigments as well as signal transduction proteins, including visual arrestin. Previous studies have indicated that the pineal pigment parapinopsin has evolutionally and physiologically important characteristics. Parapinopsin is phylogenetically related to vertebrate visual pigments. However, unlike the photoproduct of the visual pigment rhodopsin, which is unstable, dissociating from its chromophore and bleaching, the parapinopsin photoproduct is stable and does not release its chromophore. Here, we investigated arrestin, which regulates parapinopsin signaling, in the lamprey pineal organ, where parapinopsin and rhodopsin are localized to distinct photoreceptor cells. We found that beta-arrestin, which binds to stimulated G protein-coupled receptors (GPCRs) other than opsin-based pigments, was localized to parapinopsin-containing cells. This result stands in contrast to the localization of visual arrestin in rhodopsin-containing cells. Beta-arrestin bound to cultured cell membranes containing parapinopsin light-dependently and translocated to the outer segments of pineal parapinopsin-containing cells, suggesting that beta-arrestin binds to parapinopsin to arrest parapinopsin signaling. Interestingly, beta-arrestin colocalized with parapinopsin in the granules of the parapinopsin-expressing cell bodies under light illumination. Because beta-arrestin, which is a mediator of clathrin-mediated GPCR internalization, also served as a mediator of parapinopsin internalization in cultured cells, these results suggest that the granules were generated light-dependently by beta-arrestin-mediated internalization of parapinopsins from the outer segments. Therefore, our findings imply that beta-arrestin-mediated internalization is responsible for eliminating the stable photoproduct and restoring cell conditions to the original dark state. Taken together with a previous finding that the bleaching pigment evolved from a non-bleaching pigment, vertebrate visual arrestin may have evolved from a “beta-like” arrestin by losing its clathrin-binding domain and its function as an internalization mediator. Such changes would have followed the evolution of vertebrate visual pigments, which generate unstable photoproducts that independently decay by chromophore dissociation.

## Introduction

Rhodopsin and related photosensitive pigments consist of the protein moiety opsin and the chromophore retinal [1]. It is widely accepted that opsins have evolved from a non-light-sensing G protein-coupled receptor (GPCR) [2]. More than 2000 opsins have been identified in both vertebrates and invertebrates, and they are divided into several classes. Previous biochemical and spectroscopic studies have revealed that, during molecular evolution, vertebrate rod and cone visual pigments have acquired unique properties that other opsin-based pigments do not have [3]. Vertebrate visual pigments convert to a photoproduct (the meta II state), which activates G protein upon light absorption. The photoproducts of vertebrate visual pigments are unstable; they release retinal from the protein moiety, bleach and self-decay. Opsin-based pigments, including vertebrate visual pigments, that generate unstable photoproducts are called bleaching pigments. In contrast to vertebrate rod and cone pigments, many other opsin-based pigments, such as invertebrate visual pigments and melanopsins, have stable photoproducts that do not bleach by dissociation of the chromophore retinal from the protein moiety. These stable photoproducts can revert to the original dark state by subsequent light absorption [1], [4]–[6]. Opsin-based pigments, of which the photoproducts are stable and do not bleach are called non-bleaching pigments in this paper.

Recently, we clarified the molecular properties of a non-visual pineal pigment, parapinopsin, which acts as a UV-sensitive pigment and underlies wavelength discrimination in the pineal organ of lower vertebrates [7]–[8]. Parapinopsin has an amino acid sequence similar to those of vertebrate visual pigments, but our spectroscopic analysis of parapinopsin expressed in cultured cells has revealed that parapinopsin has the molecular properties of non-bleaching pigments [8] like invertebrate visual pigments and melanopsins [1], [4]–[5]. Mutational analyses of the counterion in parapinopsin (an amino acid residue essential for visible light absorption) also demonstrated the similarity of parapinopsin to non-bleaching pigments. In contrast to the Glu113 counterion in vertebrate visual pigments, parapinopsin has a Glu181 counterion like invertebrate rhodopsins, although parapinopsin has glutamic acid residues at both positions [3]. In addition, parapinopsin has much lower G-protein activation ability than vertebrate visual pigments, similar to invertebrate visual pigments [3]. Taken together, these facts suggest that vertebrate visual pigments that undergo bleaching have evolved from an ancestral, vertebrate non-bleaching pigment similar to parapinopsin. Parapinopsin, therefore, is a key pigment for understanding the evolution of vertebrate visual pigments.

In vertebrate visual cells, the light-absorbed visual pigment associates with signal transduction proteins specialized for light-sensing, e.g., visual G protein transducin and visual arrestin, which binds to the light-stimulated visual pigment to shut off G protein-mediated signaling. However, a transducin-like visual G protein or visual arrestin has not been found in the genome sequence of ascidians, which are some of the invertebrates most closely related to vertebrates. Interestingly, the ascidian arrestin binds to opsin-based pigments, a function similar to the vertebrate non-visual arrestin, β-arrestin [9]. Although these facts allow us to speculate that opsin evolution is correlated with the evolution of visual arrestin, the evolutionary link between opsin and arrestin in vertebrates is still unclear. Here we investigated arrestin, which binds to parapinopsin, using the lamprey pineal organ, in which parapinopsin-containing cells and rhodopsin-containing cells are localized in the dorsal and ventral regions of the pineal organ, respectively [8], [10]. This spatial separation allowed us to easily compare arrestins in the parapinopsin and rhodopsin systems in the same organ.

Previous studies have revealed that mammals and other lower vertebrates have two functionally different kinds of arrestins, visual arrestin and β-arrestin. Visual arrestin binds to light-stimulated visual pigments and shuts off their G protein-mediated signaling, serving as one of the key proteins in the termination of phototransduction. In arrestin knockout mice, rod cells, which lack visual arrestin, display a prolonged response to light compared to the rod cells of wild-type mice [11]. Vertebrate β-arrestin interacts with various GPCRs, but not visual pigments, *in vivo*. In various mammalian GPCR systems, β-arrestin generally has two major functions that are carried out via binding to stimulated GPCRs [12]–[16]: termination of GPCR signaling to G proteins like visual arrestin and involvement in the clathrin-mediated internalization process that removes receptors from the cell membrane to desensitize the cell. With respect to the latter function, β-arrestin has a clathrin-binding domain, which visual arrestin lacks.

In this paper, we identified two kinds of arrestins, lamprey homologues of vertebrate visual arrestin and β-arrestin, from the lamprey pineal organ using PCR. In the lamprey pineal organ, lamprey visual arrestin is localized to rhodopsin-containing photoreceptor cells, as noted previously [17], whereas lamprey β-arrestin is localized to parapinopsin-containing photoreceptor cells. This observation is the first to suggest the binding of β-arrestin, and not visual arrestin, to rhodopsin-like pigments in photoreceptor cells. We investigated the behavior of β-arrestin in parapinopsin-containing photoreceptor cells compared with that of visual arrestin in rhodopsin-containing cells in the lamprey pineal organ. We also discuss the linkage between the molecular evolution of vertebrate arrestins and photopigments.

## Results

**Figure 1:**
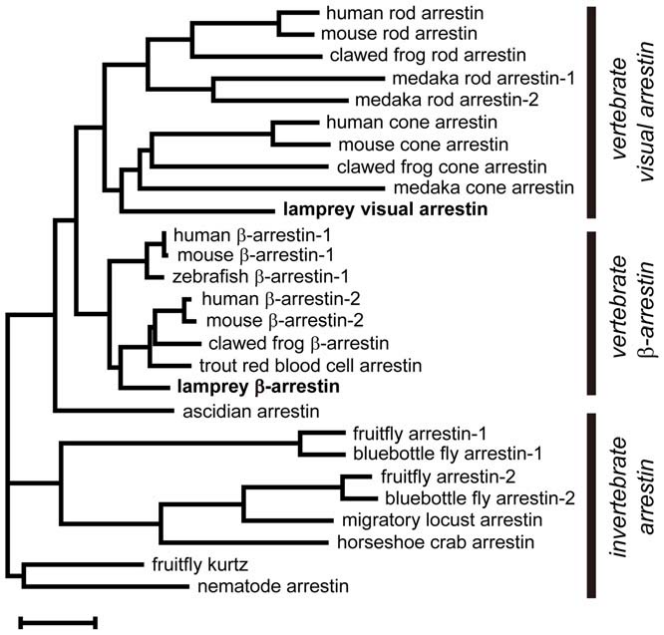
Phylogenetic positions of lamprey arrestins. The two arrestins isolated from the lamprey pineal organ were classified into the vertebrate visual arrestin and the vertebrate β-arrestin groups. Scale bar, 0.1 substitutions per site.

We isolated two arrestin cDNAs from the lamprey pineal organ by PCR amplification. Figure 1 shows the molecular phylogenetic tree of the arrestin family, including the lamprey arrestins. One arrestin (lamprey visual arrestin, AB495339) was classified as a vertebrate visual arrestin, which functions in vertebrate photoreceptor cells. The other (lamprey β-arrestin, AB495338) fell into the family of vertebrate β-arrestins, which couple to various GPCRs, excluding visual pigments.

**Figure 2:**
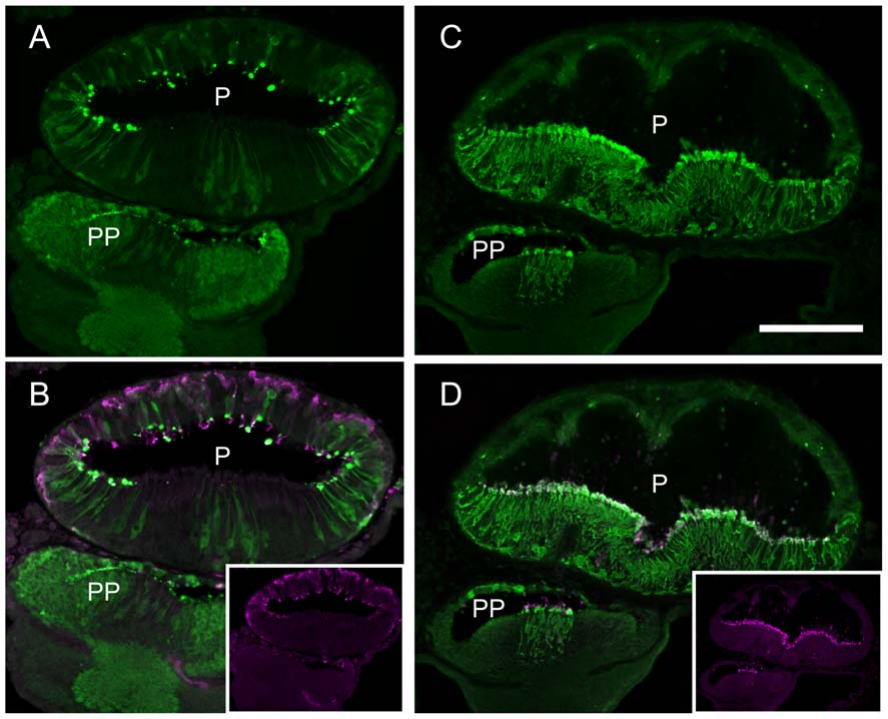
Localization of arrestins in the lamprey pineal organ. A: Immunoreactivity to lamprey β-arrestin (green); B: merged image of immunoreactivity to β-arrestin (A) and parapinopsin (inset, magenta); C: immunoreactivity to lamprey visual arrestin (green) and D: merged image of immunoreactivity to visual arrestin (C) and rhodopsin (inset, magenta). β-arrestin is localized to the dorsal photoreceptor cells (A), whereas lamprey visual arrestin is localized to the ventral photoreceptor cells (C). P, pineal organ; PP, parapineal organ. Scale bar, 100 µm.

We analyzed the localization of the arrestins in the lamprey pineal organ to investigate their functional coupling with the two kinds of photopigments. We generated antibodies against lamprey visual arrestin and β-arrestin. As shown in Figure S1, the antibodies specifically recognized visual arrestin and β-arrestin, both of which were expressed in HEK 293S cells. Therefore, we analyzed the immunohistochemical localization of visual arrestin and β-arrestin in the lamprey pineal organ using these specific antibodies (Figure 2). Visual arrestin was localized to the ventral region of the pineal organ (Figure 2C), which is consistent with previous reports [17]. Surprisingly, β-arrestin was localized to the dorsal region and showed a strong correlation with the localization of parapinopsin (Figure 2A). Therefore, we investigated the co-localization of β-arrestin and parapinopsin using a double immunostaining technique. As shown in Figure 2B, parapinopsin and β-arrestin colocalized in the dorsal photoreceptor cells of the pineal organ. This distribution pattern stands in contrast to the colocalization of visual arrestin and rhodopsin in the ventral photoreceptor cells (Figure 2D). These results imply that lamprey β-arrestin, instead of visual arrestin, interacts with light-stimulated parapinopsin to terminate the signaling from parapinopsin to G proteins. Thus, lamprey β-arrestin acts similarly to the manner in which mammalian β-arrestin is involved in the termination of signaling between stimulated GPCRs and G proteins by binding to the GPCRs [16].

**Figure 3:**
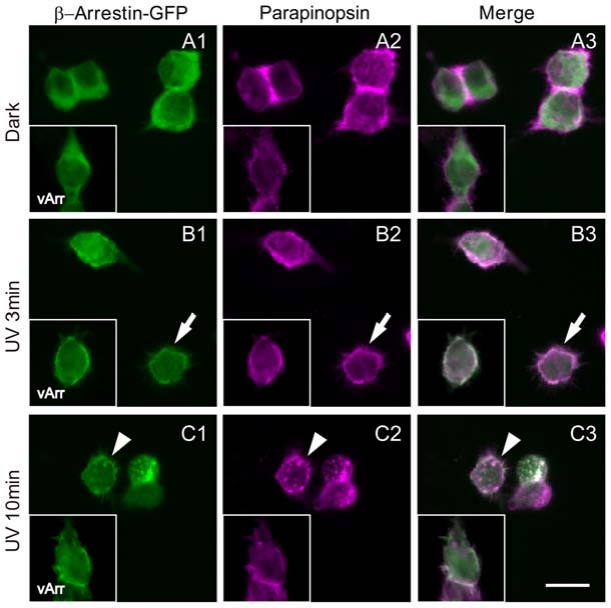
Light-induced translocation of β-arrestin with parapinopsin in cultured cells. HEK 293S cells expressing lamprey parapinopsin and lamprey β-arrestin-GFP before irradiation (A) and 3 min (B) and 10 min (C) after irradiation with UV light. Panels Ai-Ci and panels Aii–Cii show fluorescence images of β-arrestin-GFP (green) and parapinopsin immunoreactivity (magenta), respectively. The inset of each figure shows the HEK 293S cells expressing lamprey visual arrestin-GFP (Ai–Ci inset, green) and lamprey parapinopsin (Aii–Cii inset, magenta) under the same light conditions. Panels Aiii–Ciii are merged images. Note that β-arrestin-GFP translocated to the cell membrane after UV irradiation (arrows) and subsequently appeared as granules also containing parapinopsin in the cytoplasm (arrowheads). In the HEK 293S cells expressing visual arrestin-GFP and parapinopsin, visual arrestin translocated to the cell membrane but was not observed in the intracellular granules after irradiation with UV light (inset). Scale bar, 10 µm.

We also investigated the UV-induced interaction of lamprey β-arrestin with parapinopsin in cultured cells because parapinopsin has been characterized as a UV-sensitive pigment [8]. In HEK 293S cells which stably and transiently express lamprey parapinopsin and β-arrestin-GFP, respectively, parapinopsin is localized to cell membranes. β-arrestin-GFP is observed throughout the cells, except for the nuclei, but primarily localizes to the cytoplasm (Figure 3Ai–iii, Figure S2). Upon UV light irradiation, β-arrestin-GFP was detected more strongly in the cell membranes alongside parapinopsin than in the cytoplasm in about half of the cells (Figure 3Bi–iii, Figure S3), suggesting that β-arrestin bound to parapinopsin in the cell membranes. It should be noted that this clear translocation was observed in roughly half of the cells. This apparent discrepancy is likely the result of differing amounts of β-arrestin relative to parapinopsin in each cell because of β-arrestin was transiently expressed after transfection and parapinopsin was stably expressed. An *in vitro* experiment using purified bovine β-arrestin also suggested that UV-irradiated parapinopsin bound to β-arrestin (Figure S4). Interestingly, during the 10 min after UV light irradiation, we observed the gradual formation of intracellular granules in which both parapinopsin and β-arrestin-GFP were co-localized (Figure 3Ci–iii). This observation was made for about half of the cells that exhibited clear translocation of β-arrestin (Figure S3). However, the co-expression of visual arrestin with parapinopsin caused the translocation of visual arrestin to the cell membranes in response to UV light irradiation but did not result in the formation of intracellular granules during further incubation after UV light irradiation (Figure 3, insets). These observations suggest that β-arrestin plays an additional role in the formation of such intracellular granules after UV light irradiation.

It is well known that mammalian β-arrestin underlies not only the termination of GPCR signaling but also clathrin-mediated GPCR internalization, which forms granules containing both β-arrestin and internalized GPCRs [12], [15]. Therefore, we immunohistochemically investigated the involvement of clathrin in the formation of the granules. We observed a light-dependent co-localization of clathrin and parapinopsin in granules formed in HEK 293S cells (Figure S5), which is consistent with the presence of a clathrin-binding domain in lamprey β-arrestin. Therefore, like mammalian β-arrestin, lamprey β-arrestin may not only terminate parapinopsin signaling but may also promote the clathrin-mediated internalization of light-stimulated parapinopsin.

**Figure 4:**
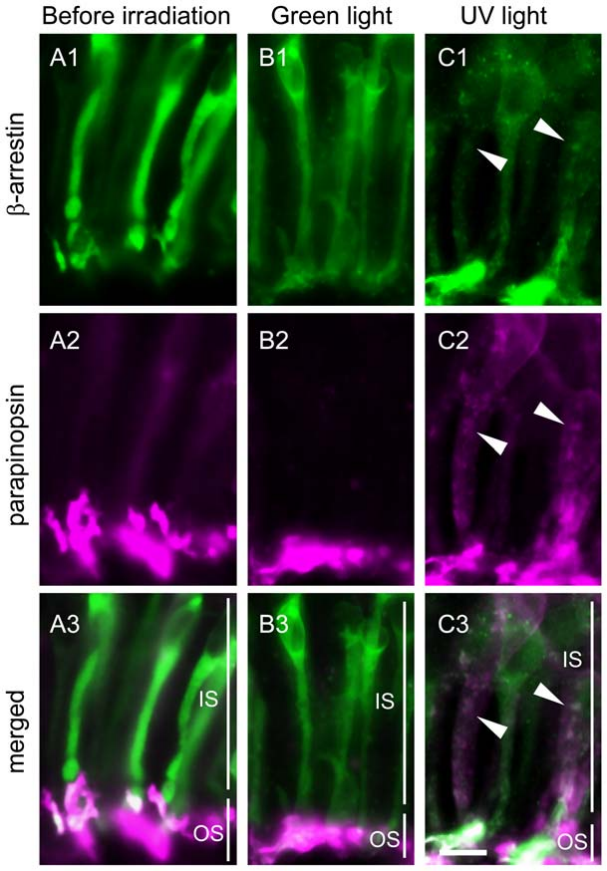
Light-regulated translocation of both β-arrestin and parapinopsin in the pineal photoreceptor cells. Parapinopsin-containing photoreceptor cells in the dorsal region of the isolated pineal organs before irradiation (A), after irradiation with green light for 6 hours (B) and after irradiation with UV light for 6 hours (C). Panels Ai–Ci and panels Aii–Cii show β-arrestin (green) and parapinopsin (magenta), respectively. Panels Aiii–Ciii are merged images of both immunoreactivities. Many granules in the inner segment were stained by antibodies to both parapinopsin and β-arrestin (arrowheads in Ci–iii). IS, inner segment; OS, outer segment. Scale bars, 10 µm.

**Figure 5:**
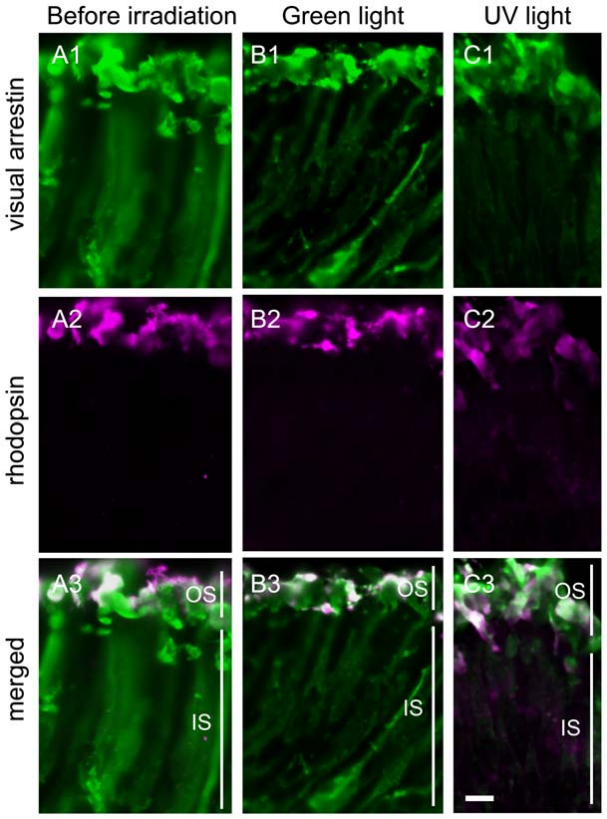
Light-regulated translocation of visual arrestin but not rhodopsin in the pineal photoreceptor cells. Rhodopsin-containing photoreceptor cells in the ventral region of isolated pineal organs before irradiation (A), after irradiation with green light for 6 hours (B) and after irradiation with UV light for 6 hours (C). Panels Ai-Ci and panels Aii–Cii show visual arrestin (green) and rhodopsin (magenta) immunoreactivities, respectively. Panels Aiii–Ciii are merged images of both immunoreactivities. The results indicate that visual arrestin translocated to the outer segments, but no granules containing rhodopsin and visual arrestin were observed. IS, inner segment; OS, outer segment. Scale bars, 10 µm.

We examined the possibility that β-arrestin was actually involved in the termination of parapinopsin signaling and parapinopsin internalization in the pineal photoreceptor cells. We compared the light-dependent translocation of β-arrestin in the parapinopsin-containing dorsal photoreceptor cells with that of visual arrestin in the rhodopsin-containing ventral photoreceptor cells. In the parapinopsin-containing photoreceptor cells before exposure of the pineal organ to light (Figure 4Ai, Figure S6A), β-arrestin was found in both the inner and outer segments of the photoreceptor cells. Under irradiation with green light, the localization profile was almost identical to the localization before light irradiation (Figure 4Bi). However, under irradiation with UV light, relatively stronger β-arrestin immunoreactivity was observed in the outer segment than in the inner segment (Figure 4Ci, Figure S6B). Because parapinopsin is a UV-sensitive non-bleaching pigment, irradiation with UV light, but not green light, was predicted to form light-stimulated parapinopsin. The formation of light-stimulated parapinopsin induced the translocation of β-arrestin to the outer segments. However, similar to β-arrestin, visual arrestin was distributed throughout the photoreceptor cells before irradiation (Figure 5Ai), and its translocation to the outer segments was observed under green light (Figure 5Bi). This translocation is very similar to the translocation reported in the rod visual cells of vertebrates [18]. These observations suggest that β-arrestin translocates and binds to light-stimulated parapinopsin in the outer segments of the pineal photoreceptor cells, similar to the light-dependent binding of visual arrestin to rhodopsin.

**Figure 6:**
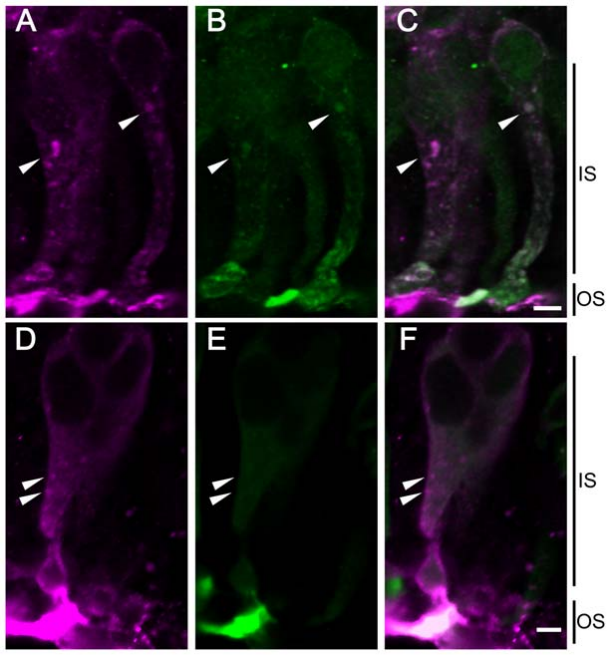
Confocal images showing localization of parapinopsin, β-arrestin and G protein under UV light. Panels A and D: Immunoreactivity to parapinopsin (magenta); panel B: immunoreactivity to β-arrestin (green); panel C: merged image of A and B; panel E: immunoreactivity to G proteins (green) and F: merged image of D and E. Parapinopsin and β-arrestin colocalized to the granules of the inner segments (arrowheads in A–C), whereas no G protein immunoreactivity was distributed in the granules of the inner segments, in which parapinopsin was localized (arrowheads in D–F). IS, inner segment; OS, outer segment. Scale bars, 10 µm.

Interestingly, after irradiation with UV light, many granules or vesicles containing β-arrestin were observed in the inner segments or cell bodies of the parapinopsin-containing photoreceptor cells (Figure 4Ci). The granules were observed in approximately 30% of the cells. In the rhodopsin-containing ventral photoreceptor cells, morphologically similar granules containing visual arrestin were not formed after irradiation with green light (Figure 5Bi) or UV light (Figure 5Ci). Double-staining experiments clearly showed that β-arrestin and parapinopsin colocalized to the granules (Figure 4Ciii), and confocal imaging of double-stained cell also confirmed their colocalization (Figure 6A–C). Together, these experiments suggest that lamprey β-arrestin is responsible for the formation of parapinopsin-containing vesicles and/or their translocation within the pineal photoreceptor cells. Granules containing both β-arrestin and parapinopsin were seen under UV light but not under green light, indicating that the formation of the granules takes place in the presence of light-stimulated parapinopsin in the photoreceptor cells.

## Discussion

In this study, we sought to identify an arrestin that interacts with the non-bleaching pigment parapinopsin. We have shown that β-arrestin colocalized with parapinopsin in pineal photoreceptor cells (Figure 2). In cultured cells, β-arrestin bound to light-stimulated parapinopsin-containing membranes (Figures 3, S2 and S3). In the parapinopsin-containing photoreceptor cells, β-arrestin was translocated to the outer segments in a light-dependent manner (Figure 4), much like visual arrestin does in rhodopsin-containing photoreceptor cells (Figure 5). These findings suggest that β-arrestin binds to light-stimulated parapinopsin to shut off signaling to G protein in the pineal photoreceptor, a function similar to the binding of visual arrestin to rhodopsin. This study is the first to observe the coupling of β-arrestin to a rhodopsin-like pigment in photoreceptor cells in a light-dependent manner.

Interestingly, β-arrestin also colocalized with parapinopsin in the granules of the inner segments or cell bodies under UV-light conditions in pineal photoreceptor cells (Figure 4C). Like mammalian β-arrestin, which serves as a mediator of clathrin-dependent GPCR internalization [13]–[15], lamprey β-arrestin may modulate the internalization of parapinopsin in HEK 293S cells in a clathrin- and light-dependent manner (Figures 3 and S5). Immunohistochemistry also showed the presence and absence of clathrin in the parapinopsin- and rhodopsin-containing photoreceptor cells, respectively, in the pineal organ (Figure S7). Therefore, we speculate that the granules were generated by a similar internalization process involving lamprey β-arrestin and clathrin. Our speculation is supported by the formation of granules under the light in the β-arrestin system (Figure 4) and not in the visual arrestin system (Figure 5).

It is of interest to discuss the physiological relevance of different kinds of arrestins and different pigment properties. The photoproduct of the visual pigment rhodopsin bleaches with time by the release of the chromophore all-*trans* retinal after transient termination through visual arrestin binding, and the uptake of 11-*cis* retinal regenerates rhodopsin. In other words, the instability and bleaching properties of the rhodopsin photoproduct are responsible for the abolishment of the photoproduct and recovery of the original dark state. However, parapinopsin converts to a photoproduct that is stable and does not bleach. Therefore, the parapinopsin photoproduct does not release the chromophore retinal or is not abolished, even under strong light [8]. In this context, parapinopsin internalization mediated by β-arrestin may play an important role in photoproduct removal after transient termination by β-arrestin binding in the course of recovery of the original dark state. Because it is widely accepted that β-arrestin-mediated internalization involves GPCRs but not G proteins [19], we speculate that the granules with parapinopsin and β-arrestin do not contain enough G proteins to function. Our immunohistochemical analysis with the antibody against transducin (TF15; Santa Cruz Biotechnology) indicated that G protein immunoreactivity was not observed in the granules, although strong immunoreactivity was seen in the outer segment (Figure 6D–F). These data support the assertion that the granules do not contain enough G proteins to function, and therefore, light-activated photoproducts in the granules cannot trigger the G protein-mediated phototransduction cascade. Moreover, a previous study reported that some internalized GPCRs undergo lysosomal degradation [20], showing the possibility that parapinopsin may be degraded in the granules. In both cases, it is suggested that β-arrestin-mediated internalization underlies the selective and complete removal of the stable photoproduct from the signal transduction locus for the eventual restoration of parapinopsin to its original “dark state” through newly synthesizing parapinopsin. In addition, the removal of photoproduct from the outer segments results in the down-regulation of parapinopsin function. This down-regulation may partially contribute to the light adaptation and desensitization of photoreceptor cells to light, similar to the down-regulation of ligand-binding GPCRs through internalization [12]–[16], [19].

The *Drosophila* visual pigment has a stable photoproduct similar to that of parapinopsin, but its amino acid sequence is largely different from parapinopsin [1]. In *Drosophila,* the visual pigment interacts with invertebrate-type arrestins (see Figure 1) to terminate signal transduction [21]. Interestingly, although invertebrate-type arrestins do not contain a clathrin-binding domain, they have been implicated in the light-induced clathrin-mediated internalization of visual pigments [22]–[24] through the interaction with another adaptor protein, AP-2 [25]. Although this internalization leads to photoreceptor cell degeneration, the primary physiological meaning of the internalization was also discussed to be maintenance and/or regulation of phototransduction [25]. Therefore, it can be speculated that the arrestin-mediated internalization of non-bleaching pigments is a general strategy for completely eliminating the light-activated pigment from the signaling cascades to restore photoreceptor cell conditions to the original dark state.

Vertebrate visual arrestins are found in a wide variety of vertebrates, including the lamprey. In most of these animals, visual arrestin is localized not only to the visual cells but also to the pineal photoreceptor cells, which contain a pigment that bleaches [26]. In other words, most visual arrestins function with bleaching pigments, regardless of their localization. This observation strongly supports the functional relationships between visual arrestin and bleaching visual pigments. Interestingly, the ascidian arrestin (Ci-arrestin, see Figure 1) has a clathrin-binding sequence and is capable of mediating internalization, similar to vertebrate β-arrestin [9]. Therefore, vertebrate visual arrestins seem to have diversified from their ancestral vertebrate “β-like” arrestin, which possesses a clathrin-binding sequence and functions as a mediator of internalization, and have evolved for function in visual cells. Therefore, one can speculate that vertebrate visual arrestin may lack a clathrin-binding domain and function as a mediator of internalization because the vertebrate visual pigments have newly acquired a bleaching property during their molecular evolution and no longer require internalization to exclude stably active photoproducts. As far as we know, this is the first strong argument that the evolution of visual pigments promoted the evolution and diversification of other signal transduction proteins and the acquisition of a phototransduction cascade that is unique to the vertebrate visual cell.

## Materials and Methods

### Animals

River lampreys, *Lethenteron japonicum* (Martens), were obtained commercially from the Ishikari River in Hokkaido, Japan and kept in aquaria with aerated and filtered water at 4–10°C under light-dark cycle conditions (L∶D = 12∶12 hours).

### Ethics statement

This experiment was approved by the Osaka City University animal experiment committee (#S0005) and complied with the Regulations on Animal Experiments from Osaka City University.

### Isolation of arrestin cDNA

Total RNA from the pineal organs was extracted using Sepasol(R)-RNA I (Nacalai Tesque) and was reverse-transcribed to cDNA using an oligo(dT) primer and Superscript III (Invitrogen). The cDNA was used as a template for PCR amplification using the following degenerate primers: Arr01F (5′- GITAYYTIGGIAARMGNGA -3′) and Arr02F (5′- AARGAIITITAYTAYCAYGGNGA -3′) for the forward primers and Arr03R (5′- TGIARIIHIACISWRCANGG -3′) and Arr04R (5′- ISWIGCIARRTTNGTRTC -3′) for the reverse primers. Annealing temperatures of 40°C or 48°C were used to obtain cDNA fragments containing the arrestin genes. Full-length cDNAs of the arrestins were obtained using 3′ RACE and 5′ RACE systems (Invitrogen).

### Phylogenetic tree inference

Multiple alignment of the amino acid sequences of arrestins, including the lamprey arrestins, was performed with XCED software [27]. Unambiguously aligned amino acid positions were subjected to phylogenetic analysis based on the neighbor-joining method [28] with a simple Poisson correction. The accession numbers of amino acid sequences used for analyses are provided in Text S1.

### Antibodies

Anti-visual arrestin, anti-β-arrestin and anti-clathrin heavy chain antisera were generated against 356 amino acids of the lamprey visual arrestin (D38–G393), 269 amino acids of the lamprey β-arrestin (V29–E297) and 439 amino acids of the lamprey clathrin heavy chain (S1147–Q1585), respectively, using the pMAL protein fusion and purification system (New England Biolabs) described previously [8], [10]. Anti-parapinopsin and anti-rhodopsin antisera were generated in a previous study from our group [10]. Anti-transducin (TF15), anti-GFP and anti-clathrin (TD.1) antibodies were purchased (Santa Cruz Biotechnology, Clontech).

### Tissue preparation

Brains, containing pineal organs and a small piece of adjacent tissue, were isolated from the animals. They were transferred into oxygenated lamprey Ringer's solution (138.6 mM NaCl, 2.82 mM KCl, 0.24 mM NaHCO_3_, and 2.07 mM CaCl_2_) and irradiated with UV (peak wavelength, 392 nm) or green light (peak wavelength, 525 nm) using light-emitting diodes for 6 hours. Before and after irradiation, the pineal organs were fixed in 4% paraformaldehyde in 0.1 M sodium phosphate buffer (PB, pH 7.4) overnight at 4°C. Each organ was cryoprotected by immersion in 0.1 M PB containing 15% or 30% sucrose, embedded in OCT compound (Sakura) and sectioned at 20 µm with a cryostat.

### Immunohistochemistry

Immunohistochemical analyses were conducted as reported previously [29] with the following modifications. In brief, tissue sections were incubated with a 0.1-N NaOH solution for 1 min and washed in 0.1 M phosphate buffered saline (pH 7.4) containing 0.3% Triton-X100 [30]. The sections were subsequently incubated with antibodies diluted 1∶500 and Alexa Fluor 488-conjugated anti-mouse or anti-rabbit IgG or Alexa Fluor 594-conjugated anti-mouse or anti-rabbit IgG (diluted 1∶500; Invitrogen) for immunofluorescent detection. We examined the stained sections under a fluorescence microscope and a confocal laser scanning microscope (Leica).

### Preparation of HEK 293S cells stably expressing parapinopsin

Parapinopsin cDNA was inserted into the expression vector pcDNA3.1 and transfected into HEK 293S cells [3]. To obtain 293S cells stably expressing parapinopsin, the transfected cells were maintained in a culture medium supplemented with 1 mg/ml Geneticin. Parapinopsin expression in the cells was examined initially by immunofluorescence, and clones were selected and expanded.

### Detection of the intracellular translocation of parapinopsin in the HEK 293S cells

Cells stably expressing parapinopsin were grown on glass coverslips and transiently transfected with β-arrestin or GFP-tagged β-arrestin cDNA (provided in Text S1) and bovine rhodopsin kinase cDNA (a generous gift from Professor David L. Farrens) using the calcium phosphate method, as previously reported [3]. To reconstitute the pigment, the transfected cells were incubated with an excess of 11-*cis* retinal in the culture medium overnight at 37°C. Cells on glass coverslips were transferred to PBS and exposed to UV light (peak wavelength, 392 nm) using light-emitting diodes at room temperature. The cells were fixed for 20 min at room temperature using 4% paraformaldehyde in 0.1 M PB with 5% sucrose. The intracellular localization of parapinopsin was detected by immunofluorescence.

## Supporting Information

Figure S1 **Immunoblot analyses showing the specificity of antibodies against lamprey visual arrestin and β-arrestin.** Lanes 1 and 2, lanes 3 and 4 and lanes 5 and 6 were stained with antibodies to GFP, lamprey β-arrestin and lamprey visual arrestin, respectively.Lanes 7 and 8 were stained with CBB. Odd and even lanes contain proteins from HEK 293S cells expressing GFP-tagged lamprey β-arrestin and visual arrestin, respectively. The results demonstrate that the antibodies specifically bind lamprey β-arrestin and visual arrestin.
(TIF)

Figure S2 **Localization of β-arrestin and parapinopsin in HEK 293S cells in the dark.** Panels A and B show fluorescence images of β-arrestin-GFP (green) and parapinopsin immunoreactivity (magenta), respectively. Panel C is a merged image. Nuclei are stained with Hoechst. β-arrestin was distributed throughout the cell except for the nucleus, whereas parapinopsin was localized to the cell membrane. Note that panels A-C are the same as panels Ai–Aiii except for Hoechst staining. Scale bar, 10 µm.
(TIF)

Figure S3 **Light-dependent translocation of β-arrestin and parapinopsin in cultured cells.** (A) Light conditions for the investigation of the light-dependent translocation of β-arrestin and parapinopsin. HEK 293S cells expressing both parapinopsin and β-arrestin-GFP were kept in the following light conditions: green-light irradiation for 3 min (phase 1), dark for 10 min (phase 2), UV light for 3 min (phase 3) and dark for 7 min (phases 4 and 5). (B) Fifty randomly selected cells were classified based on the subcellular distribution of β-arrestin-GFP. Cells exhibiting fluorescence intensity of β-arrestin-GFP staining more strongly in the cytoplasm than in the cell membranes and those staining more strongly in the cell membranes than the cytoplasm, but not in the granules, were classified as cells having cytoplasmic β-arrestin (open bars) and cells having membrane β-arrestin (gray bars), respectively. The cells showing more than 5 clear granules were distinguished as cells having granule β-arrestin (black bars). Although β-arrestin-GFP was found in the cytoplasm after irradiation with green light and dark conditions, a dramatic change was observed in half of the cells after UV irradiation; β-arrestin-GFP translocated from the cytoplasm to the cell membrane and subsequently appeared in the granules containing parapinopsin.
(TIF)

Figure S4 **β-arrestin binds to parapinopsin-containing membranes in a light-dependent manner** ***in vitro*** **.** The binding of β-arrestin was compared between parapinopsin-containing membranes under two conditions; one was kept in the dark to maintain the inactivated form (lanes –), and the other was irradiated with UV light to generate the activated form (lanes +). After collecting the membranes, they were analyzed with immunoblotting. Panels A and B were stained with antibodies against β-arrestin and parapinopsin, respectively. β-arrestin was detected with the light-activated form of parapinopsin, whereas there was no binding to the dark (inactivated) form (A), suggesting that β-arrestin binds light-stimulated parapinopsin. Panel B shows that equal amounts of parapinopsin were used in both conditions.
(TIF)

Figure S5 **Light-dependent translocation of clathrin and parapinopsin in cultured cells.** The subcellular localization of clathrin and parapinopsin in HEK 293S cells was compared between dark (A–C) and UV light conditions (D–F). Panels A and D: immunostaining with anti-clathrin antibody (TD.1, Santa Cruz Biotechnology, green); panels B and E: immunoreactivity to parapinopsin (magenta); panel C: merged image of A and B; panel F: merged image of D and E. Boxed regions are shown in higher magnification (insets). Clathrin was distributed in granules without parapinopsin throughout the cells kept in the dark. After irradiation with UV light, clathrin and parapinopsin co-localized to the granules (arrowhead), suggesting clathrin-mediated internalization of the light-stimulated parapinopsin. Scale bars, 10 µm.
(TIF)

Figure S6 **Immunohistochemical localization of β-arrestin in the pineal photoreceptor cells under dark (A) and light conditions (B).** Low magnification images indicate relatively stronger immunoreactivity to β-arrestin in the outer segment in most photoreceptor cells incubated in the UV light. IS, inner segment; OS, outer segment. The dotted traces indicate the landmark of the outer segments. Scale bars, 30 µm.
(TIF)

Figure S7 **Distribution of clathrin in pineal photoreceptor cells.** A: Immunostaining with anti-lamprey clathrin heavy chain antibody (green); B: immunoreactivity to lamprey parapinopsin (magenta) and C: merged image of immunoreactivity to clathrin and parapinopsin. Note that clathrin is localized to the parapinopsin-containing photoreceptor cells but not clearly present in the outer segment, probably due to low immunoreactivity of the lamprey clathrin heavy chain. On the other hand, the clathrin is not localized to the rhodopsin-containing photoreceptor cells. Scale bars, 10 µm.
(TIF)

Text S1 **Supplementary materials and methods.**
(DOC)
