# Supplementary figures and images for: Beta-Arrestin Functionally Regulates the Non-Bleaching Pigment Parapinopsin in Lamprey Pineal
Source: PLoS One. 2011 Jan 31;6(1):e16402. doi: 10.1371/journal.pone.0016402 (PMC3031554; doi:10.1371/journal.pone.0016402)

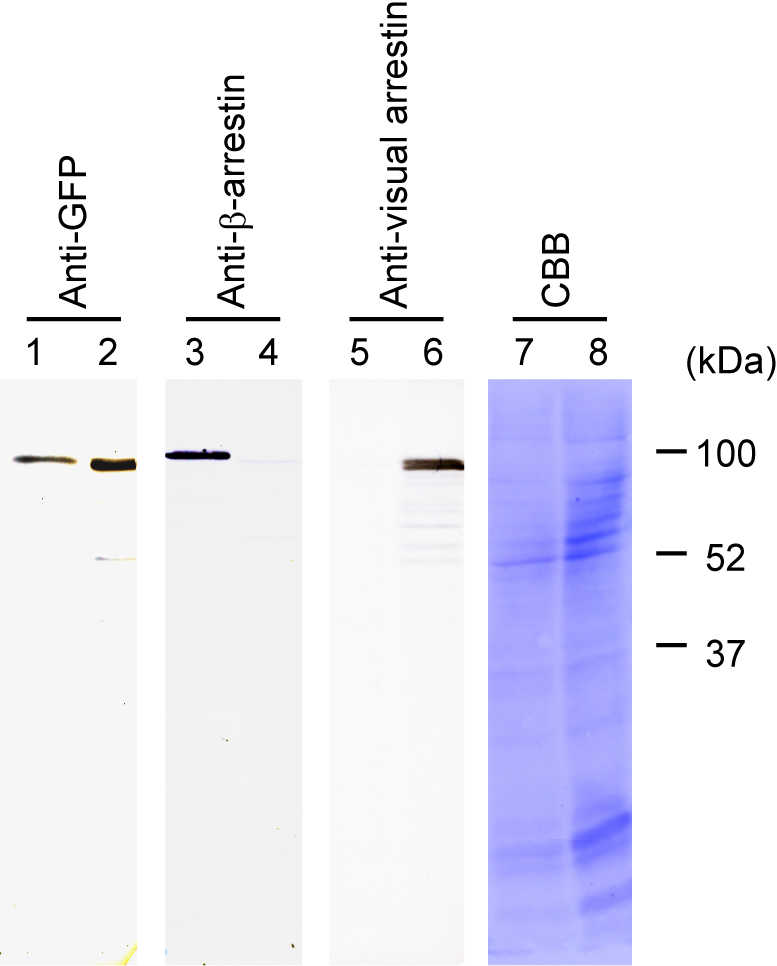

Supplement: Figure S1 — Immunoblot analyses showing the specificity of antibodies against lamprey visual arrestin and β-arrestin. Lanes 1 and 2, lanes 3 and 4 and lanes 5 and 6 were stained with antibodies to GFP, lamprey β-arrestin and lamprey visual arrestin, respectively.Lanes 7 and 8 were stained with CBB. Odd and even lanes contain proteins from HEK 293S cells expressing GFP-tagged lamprey β-arrestin and visual arrestin, respectively. The results demonstrate that the antibodies specifically bind lamprey β-arrestin and visual arrestin. (TIF) [file pone.0016402.s001.tif]

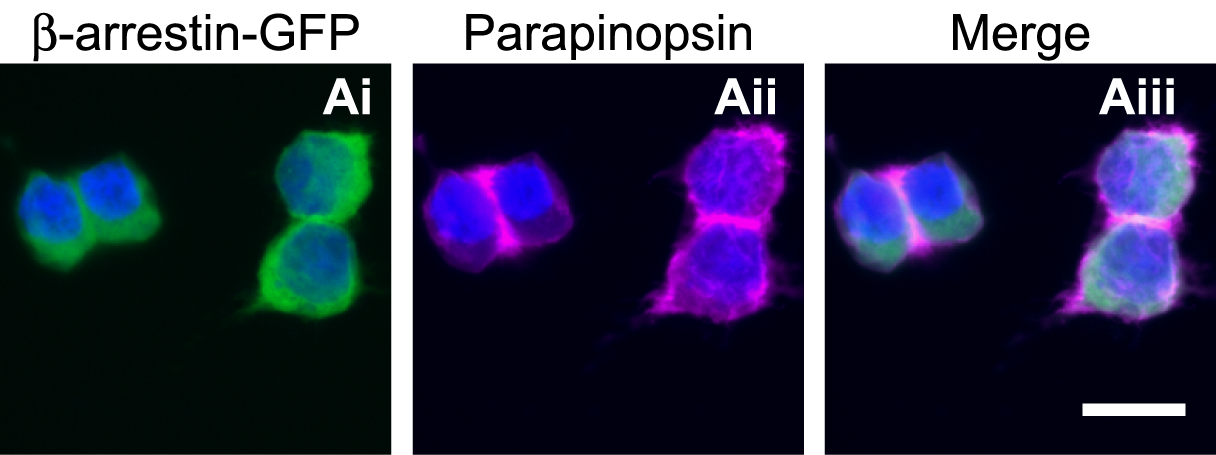

Supplement: Figure S2 — Localization of β-arrestin and parapinopsin in HEK 293S cells in the dark. Panels A and B show fluorescence images of β-arrestin-GFP (green) and parapinopsin immunoreactivity (magenta), respectively. Panel C is a merged image. Nuclei are stained with Hoechst. β-arrestin was distributed throughout the cell except for the nucleus, whereas parapinopsin was localized to the cell membrane. Note that panels A-C are the same as panels Ai–Aiii except for Hoechst staining. Scale bar, 10 µm. (TIF) [file pone.0016402.s002.tif]

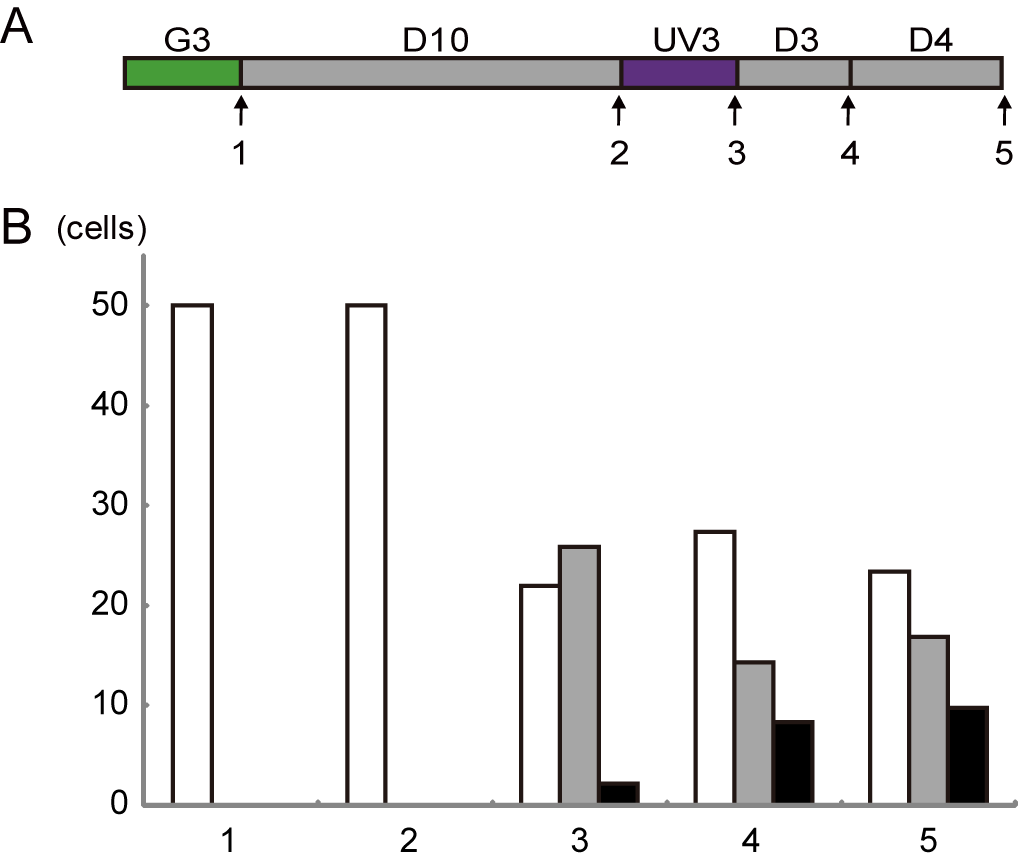

Supplement: Figure S3 — Light-dependent translocation of β-arrestin and parapinopsin in cultured cells. (A) Light conditions for the investigation of the light-dependent translocation of β-arrestin and parapinopsin. HEK 293S cells expressing both parapinopsin and β-arrestin-GFP were kept in the following light conditions: green-light irradiation for 3 min (phase 1), dark for 10 min (phase 2), UV light for 3 min (phase 3) and dark for 7 min (phases 4 and 5). (B) Fifty randomly selected cells were classified based on the subcellular distribution of β-arrestin-GFP. Cells exhibiting fluorescence intensity of β-arrestin-GFP staining more strongly in the cytoplasm than in the cell membranes and those staining more strongly in the cell membranes than the cytoplasm, but not in the granules, were classified as cells having cytoplasmic β-arrestin (open bars) and cells having membrane β-arrestin (gray bars), respectively. The cells showing more than 5 clear granules were distinguished as cells having granule β-arrestin (black bars). Although β-arrestin-GFP was found in the cytoplasm after irradiation with green light and dark conditions, a dramatic change was observed in half of the cells after UV irradiation; β-arrestin-GFP translocated from the cytoplasm to the cell membrane and subsequently appeared in the granules containing parapinopsin. (TIF) [file pone.0016402.s003.tif]

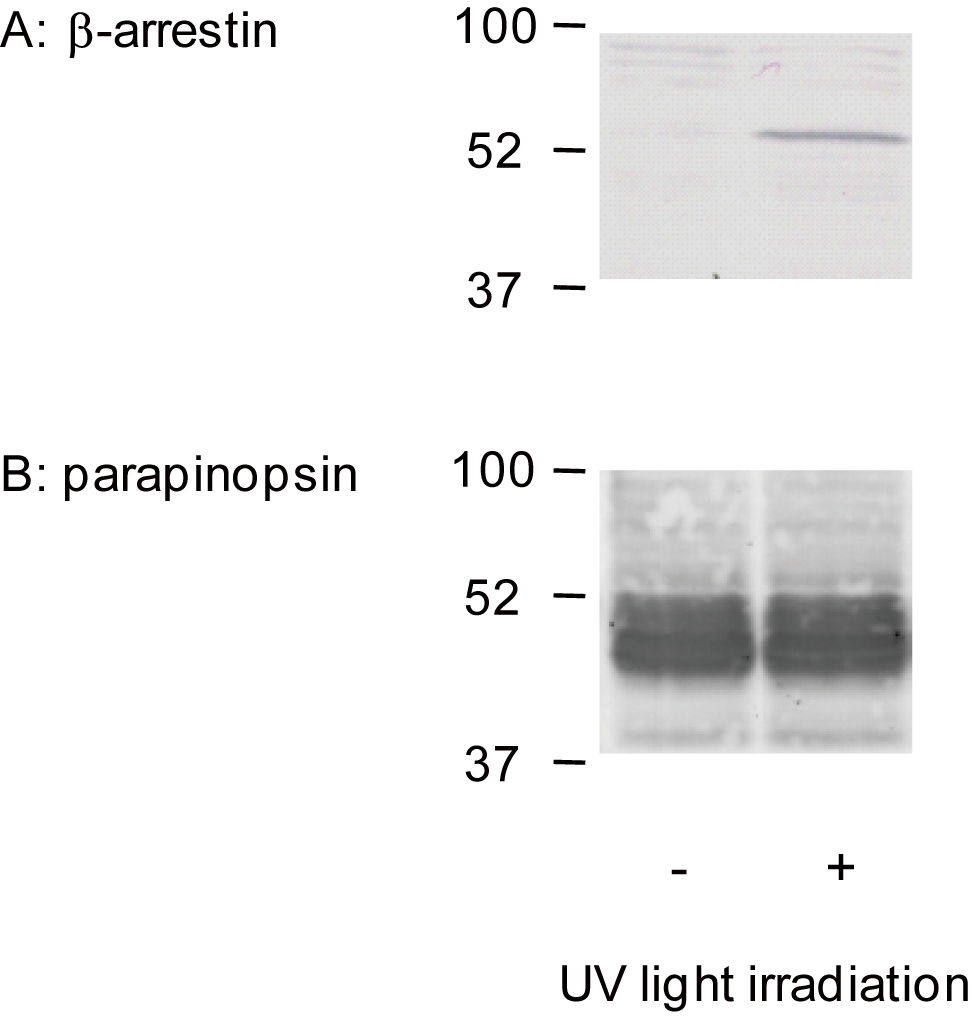

Supplement: Figure S4 — β-arrestin binds to parapinopsin-containing membranes in a light-dependent manner in vitro . The binding of β-arrestin was compared between parapinopsin-containing membranes under two conditions; one was kept in the dark to maintain the inactivated form (lanes –), and the other was irradiated with UV light to generate the activated form (lanes +). After collecting the membranes, they were analyzed with immunoblotting. Panels A and B were stained with antibodies against β-arrestin and parapinopsin, respectively. β-arrestin was detected with the light-activated form of parapinopsin, whereas there was no binding to the dark (inactivated) form (A), suggesting that β-arrestin binds light-stimulated parapinopsin. Panel B shows that equal amounts of parapinopsin were used in both conditions. (TIF) [file pone.0016402.s004.tif]

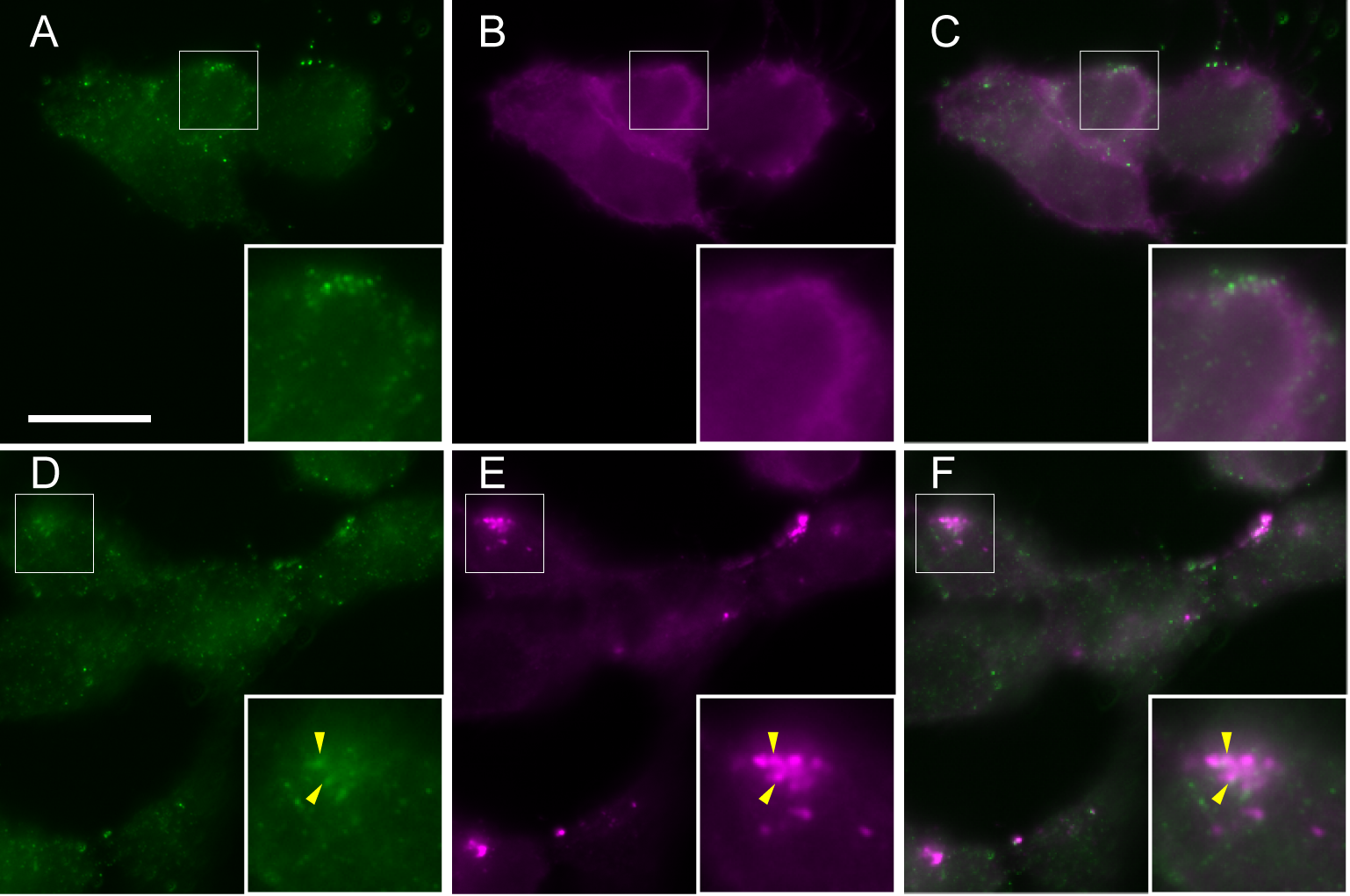

Supplement: Figure S5 — Light-dependent translocation of clathrin and parapinopsin in cultured cells. The subcellular localization of clathrin and parapinopsin in HEK 293S cells was compared between dark (A–C) and UV light conditions (D–F). Panels A and D: immunostaining with anti-clathrin antibody (TD.1, Santa Cruz Biotechnology, green); panels B and E: immunoreactivity to parapinopsin (magenta); panel C: merged image of A and B; panel F: merged image of D and E. Boxed regions are shown in higher magnification (insets). Clathrin was distributed in granules without parapinopsin throughout the cells kept in the dark. After irradiation with UV light, clathrin and parapinopsin co-localized to the granules (arrowhead), suggesting clathrin-mediated internalization of the light-stimulated parapinopsin. Scale bars, 10 µm. (TIF) [file pone.0016402.s005.tif]

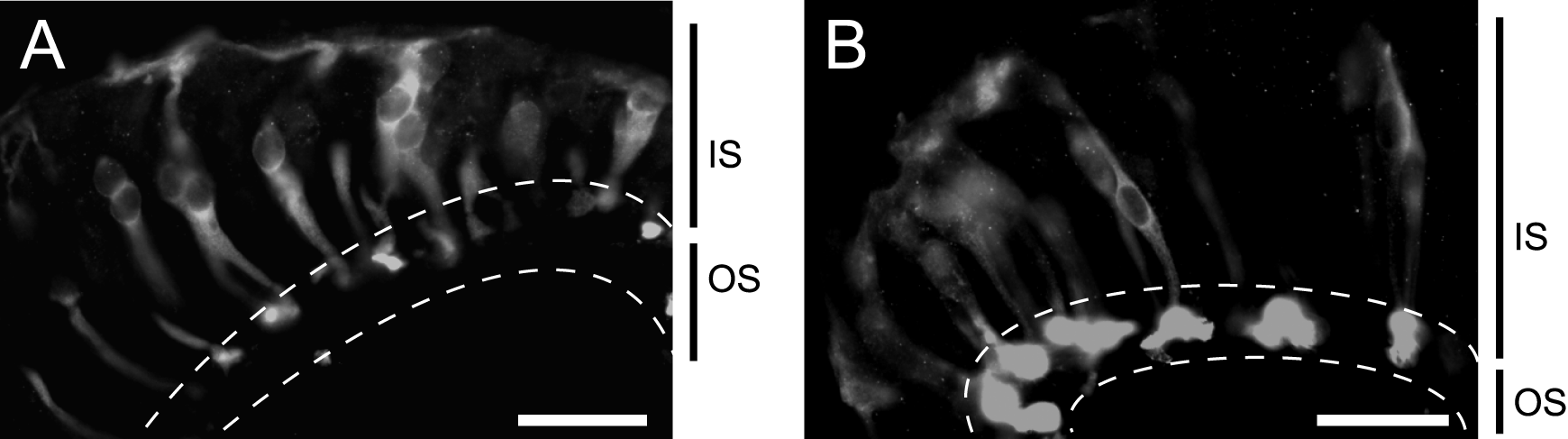

Supplement: Figure S6 — Immunohistochemical localization of β-arrestin in the pineal photoreceptor cells under dark (A) and light conditions (B). Low magnification images indicate relatively stronger immunoreactivity to β-arrestin in the outer segment in most photoreceptor cells incubated in the UV light. IS, inner segment; OS, outer segment. The dotted traces indicate the landmark of the outer segments. Scale bars, 30 µm. (TIF) [file pone.0016402.s006.tif]

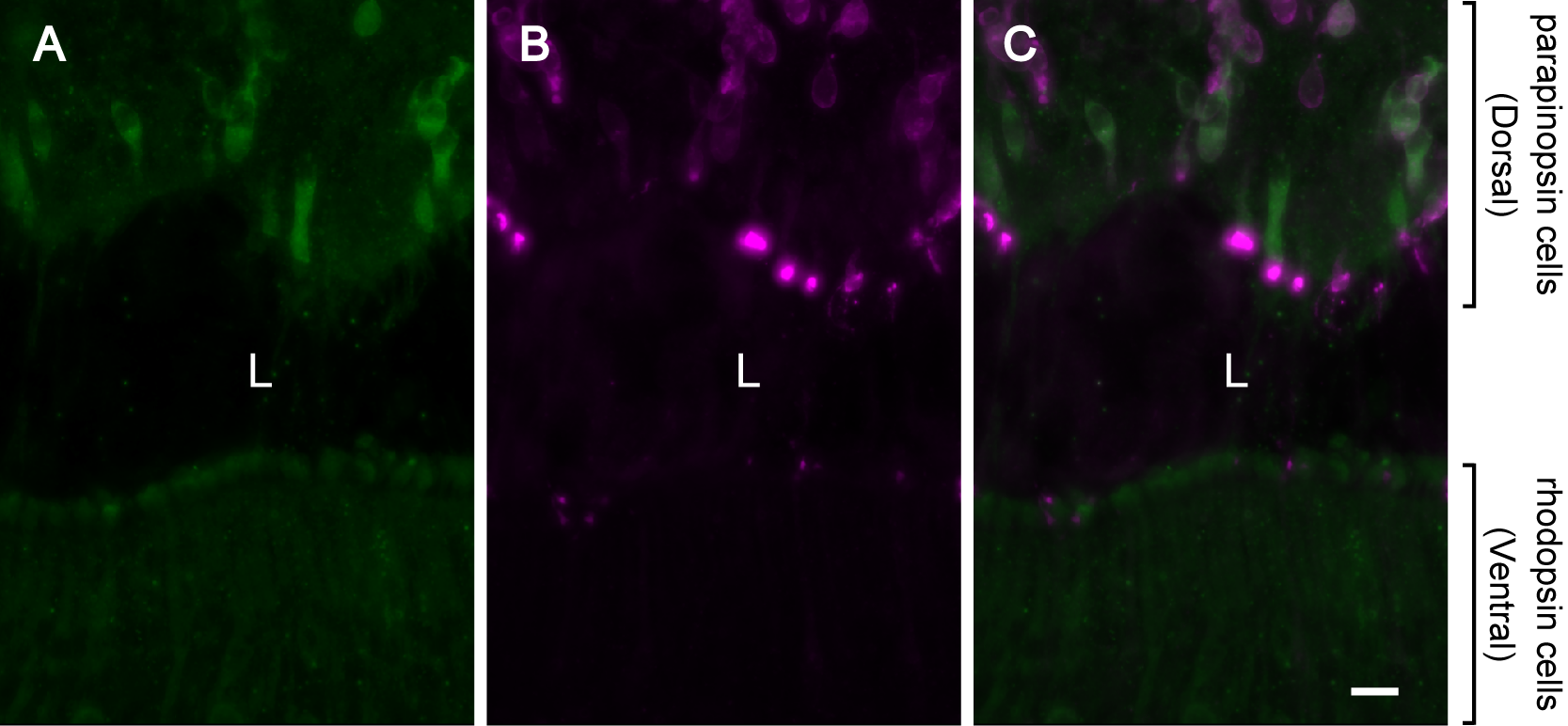

Supplement: Figure S7 — Distribution of clathrin in pineal photoreceptor cells. A: Immunostaining with anti-lamprey clathrin heavy chain antibody (green); B: immunoreactivity to lamprey parapinopsin (magenta) and C: merged image of immunoreactivity to clathrin and parapinopsin. Note that clathrin is localized to the parapinopsin-containing photoreceptor cells but not clearly present in the outer segment, probably due to low immunoreactivity of the lamprey clathrin heavy chain. On the other hand, the clathrin is not localized to the rhodopsin-containing photoreceptor cells. Scale bars, 10 µm. (TIF) [file pone.0016402.s007.tif]
