# Supplementary material for: Beta-Arrestin Functionally Regulates the Non-Bleaching Pigment Parapinopsin in Lamprey Pineal
Source: PLoS One. 2011 Jan 31;6(1):e16402. doi: 10.1371/journal.pone.0016402 (PMC3031554; doi:10.1371/journal.pone.0016402)
Supplement: Text S1 — Supplementary materials and methods. (DOC) [file pone.0016402.s008.doc]

**Text S1.**

**Supplementary Materials and Methods**

**Amino Acid Sequences.** The accession numbers of the sequence data from DDBJ/EMBL/GenBank databases are as follows: lamprey visual arrestin (AB495339), β-arrestin (AB495338); human rod arrestin (NM_000541), cone arrestin (NM_004312), β-arrestin-1 (NM_004041) and β-arrestin-2 (NM_004313); mouse rod arrestin (NM_009118), cone arrestin (NM_133205), β-arreestin-1 (NM_177231) and β-arrestin-2 (NM_145429); clawed frog rod arrestin (U41623), cone arrestin (L40463) and β-arrestin (AAH76815); medaka rod arrestin-1 (AB002554), rod arrestin-2 (AB029392) and cone arrestin (AB002555); zebrafish β-arrestin-1 (XM_001337148); rainbow trout red blood cell arrestin (U48410); ascidian arrestin (AB052668); fruitfly arrestin-1 (NM_057333), arrestin-2 (NM_079252) and kurtz (AF221066); bluebottle fly arrestin-1 (X79072) and arrestin-2 (X79073); migratory locust arrestin (S57174); horseshoe crab arrestin (U08883); nematode arrestin-like (NM_075782).

**Immunoblot analysis.** The coding regions of arrestin cDNAs were isolated by PCR with elimination of the stop codon. An equivalent PCR reaction generated an AcGFP1 (Clontech), of which the ATG initiator codon was removed. These cDNAs were inserted together into the expression vector pcDNA3.1 and used to construct arrestin-GFP. The β-arrestin cDNA was also inserted into the expression vector pcDNA3.1. The vector was transfected into HEK293S cells by the calcium phosphate method. The transfected cells were harvested for one day and collected by centrifugation. Proteins extracted from the cells were separated by SDS/PAGE, transferred onto a PVDF membrane, and incubated with antibodies to the lamprey visual arrestin (diluted 1:50000), the lamprey β-arrestin (diluted 1:5000) and GFP (diluted 1:200). Immunoreactivity was detected by the ABC kit (Vector Labs) and visualized with the horseradish peroxidase–diaminobenzidine reaction.

**β-arrestin binding to parapinopsin.** The cells expressing reconstituted parapinopsin were washed with PBS and collected by centrifugation at 1000 rpm for 3 min. Then the pellet was suspended and homogenized with 50 mM HEPES buffer (pH 6.5) containing 140mM NaCl. The cell suspension was irradiated with orange light supplied by a light source with O-53 glass cutoff filter at 5 min to generate the inactivated form of parapinopsin by photoregeneration, and the cells were collected by centrifugation at 40000 rpm for 15 min at 4 °C. Then the cell membranes were collected by sucrose flotation methods .

In the arrestin binding assay, parapinopsin was kept in the dark or exposed to UV light (a peak lambda = 392 nm) by using light-emitting diodes (LEDs) for 5 min at room temperature. Then, each form of parapinopsin was mixed in the dark with purified bovine β-arrestin (a generous gift from Professor David L. Farrens) in 50 mM HEPES buffer (pH 6.5) containing 140mM NaCl and incubated at room temperature for 10 min. The binding reaction was terminated by placement on ice and collected by centrifugation at 40000 rpm for 15 min at 4 °C. The pellets were suspended with buffer containing 10mM HEPES (pH 7.2) and centrifuged at 40000 rpm for 15 min at 4 °C. This washing step was performed twice. These samples were subjected to immunoblotting with antibodies against β-arrestin (diluted 1:5000) and parapinopsin (diluted 1:1000).

**Supplemental Reference**

1. Kojima D, Oura T, Hisatomi O, Tokunaga F, Fukada Y, et al. (1996) Molecular properties of chimerical mutants of gecko blue and bovine rhodopsin. Biochemistry 35: 2625-2629.
